# Synthesis and Characterization of an α-Fe_2_O_3_-Decorated g-C_3_N_4_ Heterostructure for the Photocatalytic Removal of MO

**DOI:** 10.3390/molecules27041442

**Published:** 2022-02-21

**Authors:** Rooha Khurram, Zaib Un Nisa, Aroosa Javed, Zhan Wang, Mostafa A. Hussien

**Affiliations:** 1 Beijing Key Laboratory for Green Catalysis and Separation, Department of Chemistry and Chemical Engineering, Beijing University of Technology, Beijing 100124, China; khurramrooha@emails.bjut.edu.cn; 2 Department of Chemistry, School of Natural Sciences (SNS), National University of Sciences and Technology (NUST), H-12, Islamabad 44000, Pakistan; zaibunisadab@gmail.com; 3 Department of Chemistry, University of Calgary, Calgary, AB T2N 1N4, Canada; aroosa.899@gmail.com; 4 Department of Chemistry, Faculty of Science, King Abdulaziz University, Jeddah P.O. Box 80203, Saudi Arabia; 5 Department of Chemistry, Faculty of Science, Port Said University, Port Said 42521, Eygpt

**Keywords:** g-C_3_N_4_, g-C_3_N_4_/α-Fe_2_O_3_ nanocomposite, MO photodegradation, heterostructure (type-II), alignment of energy levels

## Abstract

This study describes the preparation of graphitic carbon nitride (g-C_3_N_4_), hematite (α-Fe_2_O_3_), and their g-C_3_N_4_/α-Fe_2_O_3_ heterostructure for the photocatalytic removal of methyl orange (MO) under visible light illumination. The facile hydrothermal approach was utilized for the preparation of the nanomaterials. Powder X-ray diffraction (XRD), Scanning electron microscopy (SEM), Energy dispersive X-ray (EDX), and Brunauer–Emmett–Teller (BET) were carried out to study the physiochemical and optoelectronic properties of all the synthesized photocatalysts. Based on the X-ray photoelectron spectroscopy (XPS) and UV-visible diffuse reflectance (DRS) results, an energy level diagram vs. SHE was established. The acquired results indicated that the nanocomposite exhibited a type-II heterojunction and degraded the MO dye by 97%. The degradation ability of the nanocomposite was higher than that of pristine g-C_3_N_4_ (41%) and α-Fe_2_O_3_ (30%) photocatalysts under 300 min of light irradiation. The formation of a type-II heterostructure with desirable band alignment and band edge positions for efficient interfacial charge carrier separation along with a larger specific surface area was collectively responsible for the higher photocatalytic efficiency of the g-C_3_N_4_/α-Fe_2_O_3_ nanocomposite. The mechanism of the nanocomposite was also studied through results obtained from UV-vis and XPS analyses. A reactive species trapping experiment confirmed the involvement of the superoxide radical anion (O_2_^•−^) as the key reactive oxygen species for MO removal. The degradation kinetics were also monitored, and the reaction was observed to be pseudo-first order. Moreover, the sustainability of the photocatalyst was also investigated.

## 1. Introduction

Although synthetic dyes provide vibrant colors, they also cause serious water pollution problems. Dye wastewater produced by textile, paper, leather, and other industries has become one of the main sources of water pollution [1]. Among the synthetic dyes, anionic azo dyes account for half of dye synthesis and industrial application [2]. Due to their low coloring rate on natural fibers, anionic dyes account for a large proportion of the dye wastewater discharged by printing and dyeing factories. Methyl orange (MO) is a common and typical azo anionic dye. This water-soluble organic synthetic dye has very high colorability and presents a bright orange color when dissolved in water. Azo dyes, such as methyl orange, contain aromatic and –N = N– groups in their molecules, which are highly toxic, carcinogenic, and teratogenic [3,4], and are harmful to the environment and organisms [5]; thus, wastewater must be treated innocuously before it can be discharged. MO was selected as a model pollutant in this study.

In recent years, various semiconductor-based photocatalysts have been designed to perform photocatalytic tasks, including H_2_ production, CO_2_ reduction, dye degradation, etc. [6]. Despite several attempts at improvement, their performances are not satisfactory owing to the weak separation of the light-generated charge carriers with limited light-harvesting efficiency. In 2009, one of the most well-known metal-free polymeric photocatalysts, called g-C_3_N_4_, was employed in H_2_ production via water splitting by Wang et al. [7]. Afterward, this photocatalyst earned enormous attention in CO_2_ reduction and pollutant degradation, owing to suitable bandgap (2.6–2.7 eV) and band edge positions, chemical stability, and cost-effectivity [8,9,10,11,12,13]. However, the efficiency of pristine g-C_3_N_4_ is unacceptable due to its poor visible light absorption and high charge carrier recombination [14]. Various strategies, including composite formation, doping, and utilizing any photosensitizer, have been employed to address these issues [15,16]. Hematite (α-Fe_2_O_3_) is considered a promising n-type semiconductor, exhibiting a suitable band potential for efficient light absorption at a wide range of wavelengths [17]. Moreover, α-Fe_2_O_3_ possesses special characteristics, including tremendous stability, non-toxicity, photocurrent and corrosion resistance, etc. [18]. Therefore, two-component g-C_3_N_4_ based systems are synthesized to form heterojunction structures with a higher photocatalytic efficiency utilizing a wide wavelength range [19]. Thus far, various reports have published the Z-scheme action and heterojunction mechanism of the g-C_3_N_4_/α-Fe_2_O_3_ composite in pollutant degradation [20], CO_2_ reduction [21,22,23], photoelectrochemical [24,25], Hg (II) reduction [26], etc. However, there are few reports comprising the ternary and quaternary systems of g-C_3_N_4_/α-Fe_2_O_3_ for the degradation of wastewater dyes following the heterojunction mechanism [20]. The charge transfer between the interfacial phase in potential photocatalytic mechanisms may follow the typical heterojunction mechanism or Z-scheme mechanism. In the typical heterojunction mechanism, the sample absorbs visible light, which stimulates the migration of electrons from the VB to the CB, leaving h^+^ in the VB. Subsequently, h^+^ from constituent-1 migrates to the E_VB_ of constituent-2, and e^−^ from constituent-2 transfers to the E_CB_ of constituent-1. Different from the typical heterojunction photogenerated electrons and photogenerated holes, the e^−^ generated in the CB of constituent-1 moves directly to combine with the h^+^ in the VB of constituent-2. The photogenerated e^−^ in the CB of constituent-2 participates in the reduction reaction, while the h^+^ generated in the VB of constituent-1 participates in the oxidation of water. Thus, the electron transfer pathway presents a Z-shaped path. As a result of both potential photocatalytic mechanisms, the efficiency of electron-hole transfer and separation are promoted and the recombination rates of photoexcited electron-hole pairs in both constituents themselves are inhibited. However, the redox potential values with respect to the conduction and valence band position of photocatalysts play a critical role in determining the type of potential photocatalytic mechanisms. For instance, in their first report, Xu. Q. et al. investigated the superior photocatalytic performance of 2D/2D α-Fe_2_O_3_/g-C_3_N_4_ for H_2_ generation through the Z-scheme mechanism [27]. In another report, Jiang, Z. et al. developed a hierarchical Z-scheme over an α-Fe_2_O_3_/g-C_3_N_4_ hybrid for enhanced CO_2_ reduction [28]. In both these studies, the authors investigated the Z-scheme for efficient photocatalytic performance. However, the authors did not investigate the involvement of reactive oxygen species that are involved in photocatalytic activity. Recently, Mohsen Padervand et al. [29] studied the formation of ROS where ∙OH radicals were fundamentally involved in RhB degradation under light, suggesting a Z-scheme mechanism.

Owing to insufficient literature on the mechanistic investigation of type-II heterostructures to better understand ROS involvement, this novel work chose to investigate their formation by conducting an active species trapping experiment. The synthesis of an efficient g-C_3_N_4_/α-Fe_2_O_3_ heterostructure was conducted via a facile hydrothermal approach utilizing cost-effective precursors. The photocatalytic efficiency was estimated by the photodegradation of methyl orange (MO) dye under visible light illumination. The reactive species trapping experiment revealed the formation of superoxide radical anions O_2_^•−^ as primary species for MO removal. Moreover, kinetic studies were conducted to determine the order of the reaction. The results of XPS and UV-vis spectroscopy were utilized for drawing the band alignment vs. SHE. The band diagram illustrating the band edge position was developed and elaborated.

## 2. Results and Discussion

### 2.1. Physiochemical and Optoelectronic Properties of All the Synthesized Photocatalysts

**Figure 1:**
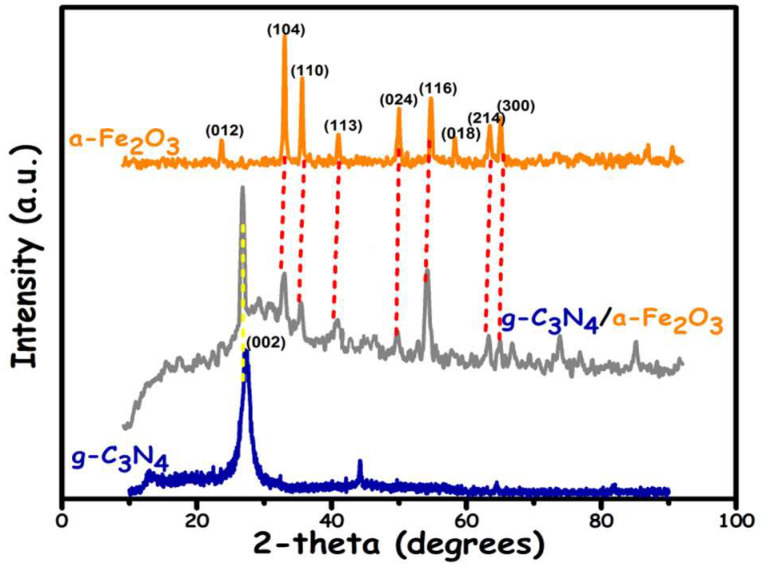
The XRD patterns.

Typical XRD patterns of g-C_3_N_4_, α-Fe_2_O_3,_ and g-C_3_N_4_/α-Fe_2_O_3_ are displayed in Figure 1. The XRD pattern of pure g-C_3_N_4_ exhibits an intense, broad, asymmetric, and characteristic peak at 27.4° indexed as the (002) diffractions for the graphitic interlayer stacking of the conjugated aromatic ring. A less intense peak at a lower angle of 13.1° was indexed as the (100) diffractions for the inter-planar stacking peaks of the tri-s-triazine units. Both the corresponding peaks perfectly matched with the JCPDS no. 87–1526. g-C_3_N_4_ was found to have a hexagonal crystal structure [30,31]. Pure α-Fe_2_O_3_ exhibited diffraction peaks at Bragg’s angle 24.2°, 33.2°, 35.6°, 40.9°, 49.5°, 54.1°, 58°, 62°, 63° indexed as (012), (104), (110), (113), (024), (116), (018), (214), and (300) diffractions, respectively, which were perfectly in accordance with the JCPDS card # 01-089-0598 for its rhombohedral crystal system [32]. In addition, the peaks of both the individual constituents, i.e., g-C_3_N_4_ and α-Fe_2_O_3_, could be seen in the XRD pattern of the g-C_3_N_4_/α-Fe_2_O_3_ composite_,_ indicating the successful in-situ synthesis of the nanocomposite and thereby endorsing the phase purity. Moreover, in the g-C_3_N_4_/α-Fe_2_O_3_ XRD pattern, no obvious peak shifting occurred, indicating that the crystal structure was maintained during the synthesis process.

The crystallite size was also calculated from the XRD results. The estimated average crystallite sizes of g-C_3_N_4_/α-Fe_2_O_3_, g-C_3_N_4_, and α-Fe_2_O_3_ were 60.5 nm, 29.4 nm, and 32.5 nm, respectively, calculated using Equation (1). Crystallite size has been recognized as an important parameter that influences the photocatalytic performance of the material [33]. The recombination of the photogenerated charge carriers by the photocatalyst sample critically depends on its crystallite size [34]. The charge carrier recombination process may be carried out in two ways: volume recombination or surface recombination [35]. Surface recombination is the dominant process in smaller crystallites. For pure/bare samples (e.g., g-C_3_N_4_ and α-Fe_2_O_3_), the charge carrier’s mobility becomes extremely low and undergoes recombination before it can reach the surface. Both g-C_3_N_4_ and α-Fe_2_O_3_ exhibit a small crystallite size, i.e., 29.4 nm and 32.5 nm, respectively; therefore, most of the charge carriers are generated sufficiently close to the surface. As a result, the photogenerated charge carriers that reach the surface result in faster recombination. This is also owing to the lack of driving force to separate the charge carriers. Further interfacial charge transfer processes will be outweighed by the surface recombination rate for smaller crystallites [35]. However, the g-C_3_N_4_/α-Fe_2_O_3_ nanocomposite exhibits a larger crystallite size, i.e., 60.5 nm. In this case, a driving force to separate the charge carriers exists. Thereby, a reduction in this surface recombination results in a reduced recombination rate of the photogenerated charge carriers and hence results in greater efficiency. Thus, a higher photocatalytic activity is observed for the nanocomposite.

**Figure 2:**
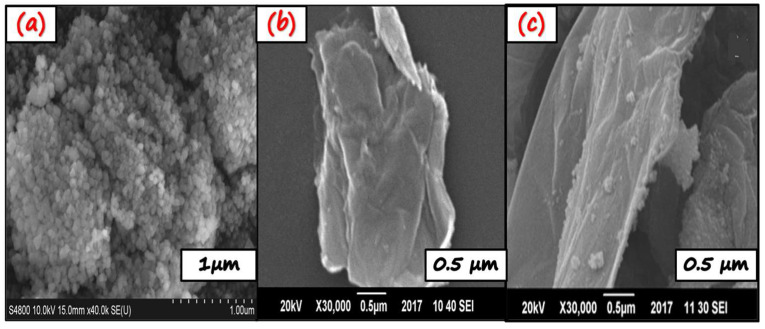
SEM micrographs of (**a**) α-Fe_2_O_3_ agglomerated nanoparticles, (**b**) g-C_3_N_4_ crumpled nanosheet, and (**c**) g-C_3_N_4_/α-Fe_2_O_3_ nanosheet well decorated with nanoparticles.

The morphological analysis was carried out by scanning electron microscopy, and the results are displayed in Figure 2. The SEM micrograph displays the laminar nanosheet-like structure of pure g-C_3_N_4_ and the agglomerated nanoparticle-like structure of α-Fe_2_O_3_. A laminar-nanosheet like morphology provides abundant active sites and space for the attachment of α-Fe_2_O_3_. Figure 2c shows the morphology of the nanocomposite. The g-C_3_N_4_ nanosheet was fully and randomly decorated with α-Fe_2_O_3_ nanoparticles. This close and strong interaction may have been established between g-C_3_N_4_ and α-Fe_2_O_3_, which facilitate the charge carriers’ separation and transfer for an improved photocatalytic response. However, in the future, this should be further confirmed through HR-TEM analysis.

**Figure 3:**
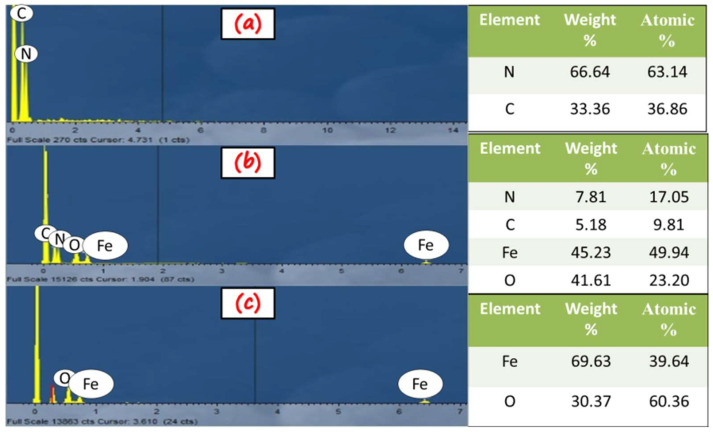
EDS spectra of: (**a**) g-C_3_N_4_, (**b**) α-Fe_2_O_3_, and (**c**) g-C_3_N_4_/α-Fe_2_O_3_ with quantification of atomic percentages.

The composition and elemental distribution of the synthesized photocatalysts were investigated by EDS, and the results are presented in Figure 3. The EDS analysis also confirmed the purity of all the synthesized samples. The EDS spectra of g-C_3_N_4_ indicated carbon and nitrogen as primary elements, as shown in Figure 3a. Figure 3c shows the distinct peaks of Fe and O for the α-Fe_2_O_3_ sample. The EDS spectrum (Figure 3b) demonstrated the distribution of C, N, Fe, and O elements without any impurity, confirming the phase purity of the synthesized α-Fe_2_O_3_/g-C_3_N_4_ nanocomposite, as supported by the XRD patterns. The weight percentage and atomic percentage of all the samples are also depicted in Figure 3.

**Figure 4:**
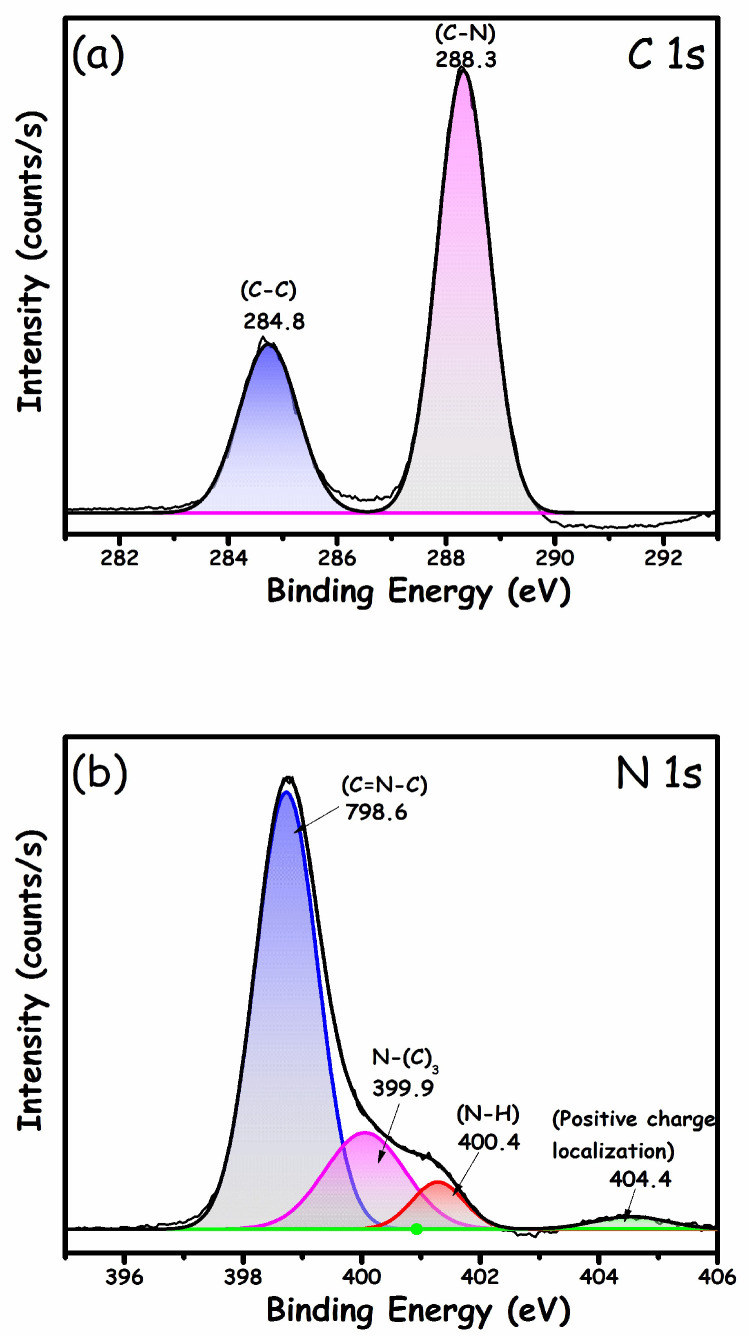

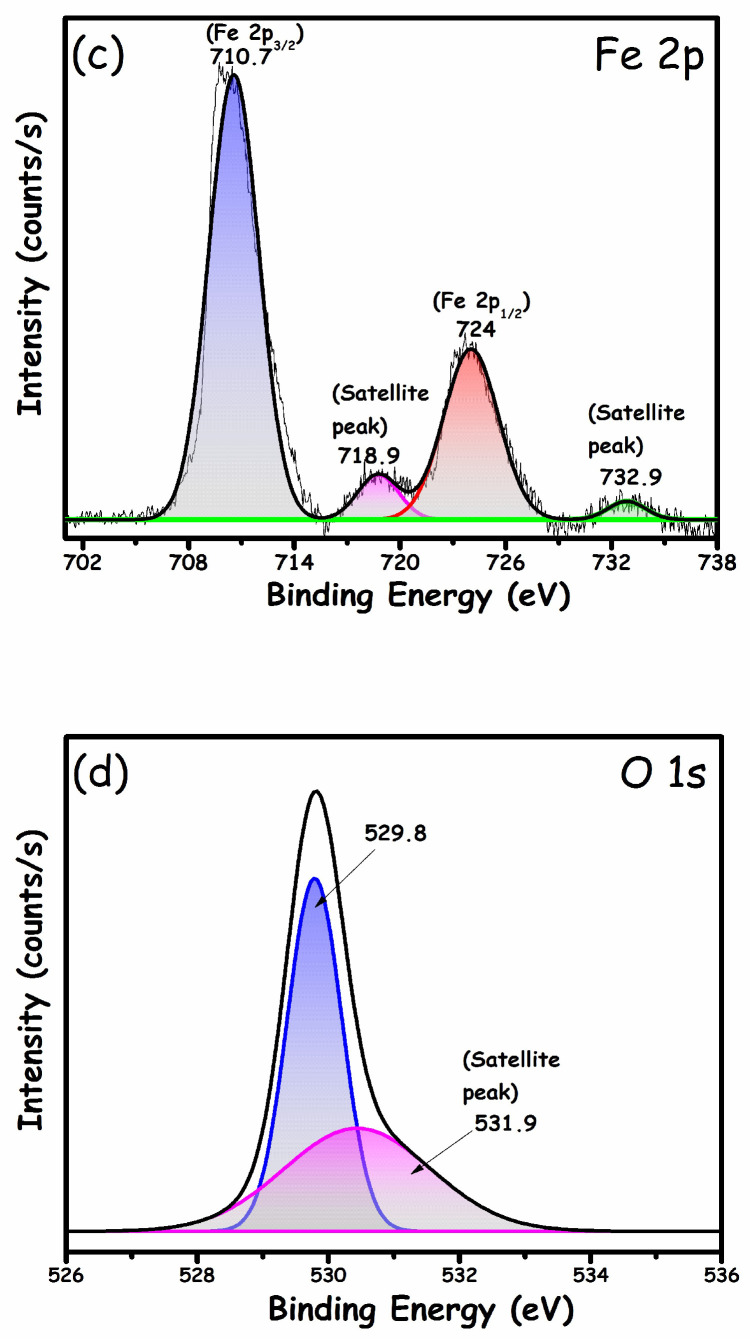
XPS spectra of g-C_3_N_4_/α-Fe_2_O_3_ nanocomposite: (**a**) C (1s), (**b**) N (1s), (**c**) Fe (2p), and (**d**) O (1s).

XPS analysis was performed to examine the surface chemistry, elemental composition, and electronic states of all the elements, and the outcomes are depicted in Figure 4. Figure 4a depicts the C 1s spectrum comprising two peaks. The peak located at 284.8 eV was attributed to sp^2^ hybridized C-C, while the second peak at 288.3 eV was attributed to sp^2^ hybridized C atoms in the aromatic ring (N=C-N) [36,37]. On the contrary, the deconvoluted N 1s spectrum (Figure 4b) depicted four distinct peaks at 398.6, 399.9, 400.4, and 404.4 eV, which were ascribed to sp^2^ hybridized N in the triazine ring (C=N-C groups), tertiary N-atoms bonded to carbon (N-(C)_3_ groups), the amino group of N-H, and the charging effect in the heterocycles, respectively [38,39]. The typical α-Fe_2_O_3_ spectra (Figure 4c) showed two distinct peaks at 710.7 and 724 eV corresponding to Fe 2p_3/2_ and Fe 2p_1/2_, respectively. Two shake-up satellite peaks characteristic of the 3^+^ oxidation state of Fe in α-Fe_2_O_3_ following each distinct peak at 718.9 and 732.9 eV were also seen_._ The O 1s spectra (Figure 4d) showed a distinct peak at 529.5 eV and a shake-up satellite peak at 531.9 eV, corresponding to the crystal lattice 2^-^ oxygen and a surface hydroxyl group, respectively [40].

**Figure 5:**
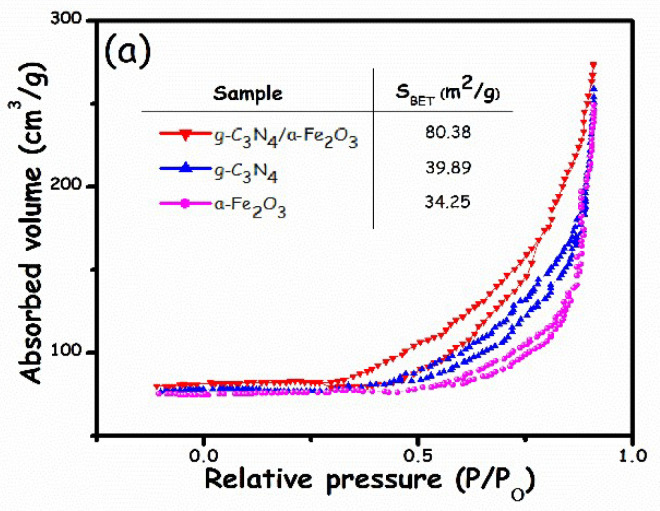

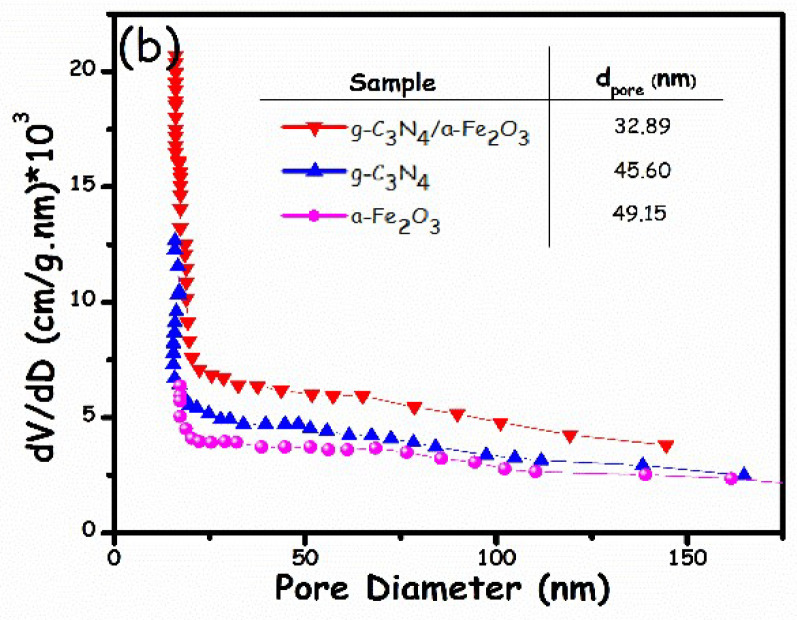
(**a**) N_2_ adsorption–desorption isotherms and (**b**) pore size distributions of α-Fe_2_O_3_, g-C_3_N_4_, and α-Fe_2_O_3_/g-C_3_N_4_ samples.

Surface area critically affects photocatalytic performance. Therefore, specific surface area measurements and pore size were investigated through N_2_ adsorption–desorption isotherms (Figure 5). All sample exhibited type IV isotherms. The results demonstrated that the surface area of the g-C_3_N_4_/α-Fe_2_O_3_ nanocomposite was significantly larger than its individual constituents, i.e., α-Fe_2_O_3_ and g-C_3_N_4_. However, the surface area of the g-C_3_N_4_ nanosheets was higher than that of α-Fe_2_O_3_. This might be attributed to the laminar sheet-like morphology of g-C_3_N_4_ or the aggregation of α-Fe_2_O_3_ nanoparticles, which lowers its surface area. From greatest to smallest, the surface areas of the prepared photocatalysts were as follows: α-Fe_2_O_3_/g-C_3_N_4_ > g-C_3_N_4_ > α-Fe_2_O_3_ (Figure 5a). However, the literature reveals conflicting results regarding the surface area analysis of the combination of α-Fe_2_O_3_ and g-C_3_N_4_. For example, Li et al. [41] synthesized an α-Fe_2_O_3_/g-C_3_N_4_ nanocomposite by the pyrolysis of melamine, and ferric nitrate showed an increment in surface area compared to pure g-C_3_N_4_. However, Sun et al. [42] prepared an α-Fe_2_O_3_/g-C_3_N_4_ nanocomposite by using precursors, including ferric chloride and dicyandiamide. Their results were antagonistic to the traditional trend: the surface area of pure g-C_3_N_4_ was reduced. Zhang et al. [43] reported no noticeable change in the surface area of g-C_3_N_4_ when the α-Fe_2_O_3_/g-C_3_N_4_ nanocomposite was synthesized by the direct mixing of α-Fe_2_O_3_ and g-C_3_N_4_. This indicates that the nanocomposite synthesis procedure plays a dominating role in determining its textural properties that influence its surface area. In this study, the surface area of the nanocomposite increased to 80.38 m^2^/g, almost one-fold greater than pristine g-C_3_N_4_. On the other hand, the surface area measurements for the g-C_3_N_4_ and α-Fe_2_O_3_ were 39.89 m^2^/g and 34.25 m^2^/g, respectively. Our findings are in good agreement with the reported studies of Li et al. [41]. A larger surface area increases the available active sites and effectively promotes adsorption and desorption, thereby enhancing the photocatalytic response. The relevant pore diameter distribution the of samples exhibited a broad distribution between 10 and 50 nm, which is characteristic of mesopores (Figure 5b). The broad distribution of pores included small and large mesopores. The smaller pores indicate the nanoporous structure on the surface of g-C_3_N_4_ nanosheets and other nanoparticles, and the larger pores are related to those formed from randomly stacked layers of graphitic carbon nitride. The porous structure should facilitate the fast transmission of reactants and products during the photocatalytic reaction process.

### 2.2. Photocatalytic Performance for MO Degradation

**Figure 6:**
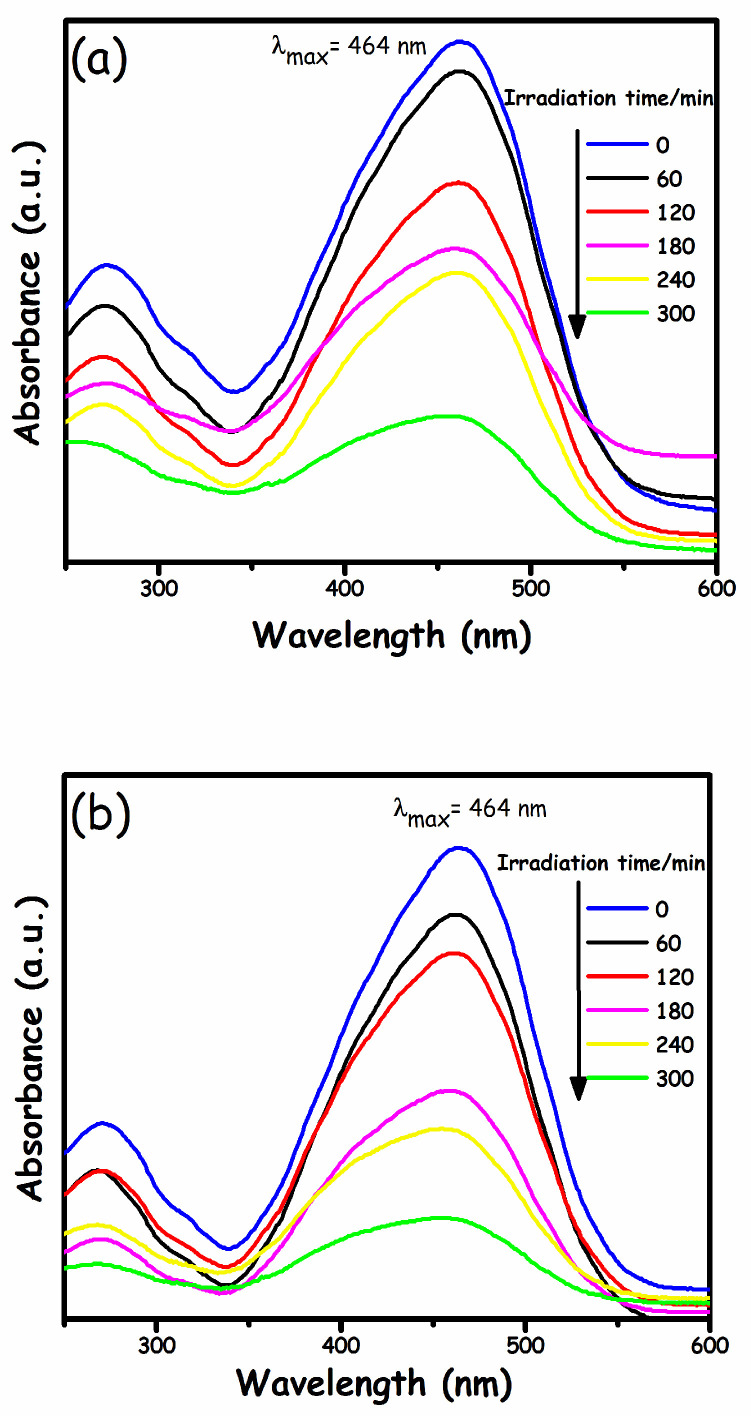
UV-vis absorption spectral changes of MO with time in (**a**) pure g-C_3_N_4_ and (**b**) g-C_3_N_4_/α Fe_2_O_3_ nanocomposite under light illumination.

The activity of the g-C_3_N_4/_α-Fe_2_O_3_ nanocomposite was evaluated by the photodegradation of MO under light. Figure 6 demonstrates the comparative analysis of spectral changes over pristine g-C_3_N_4_ and the g-C_3_N_4_/α-Fe_2_O_3_ nanocomposite. For pure g-C_3_N_4_ (Figure 6a), the intensity of the maximum absorption peak (λ_max_) of MO at 464 nm decreased slowly, indicating that the degradation rate of MO was relatively slow, which signified the presence of non-degraded MO molecules even after 300 min of reaction time. However, the g-C_3_N_4_/α-Fe_2_O_3_ nanocomposite (Figure 6b) peak at λ_max_ decreased gradually within 300 min, indicating its superior photocatalytic performance. The spectral change over the time for pure g-C_3_N_4_ and the g-C_3_N_4_/α-Fe_2_O_3_ nanocomposite was correlated with its photocatalytic performance. The photocatalytic degradation ability of g-C_3_N_4_/α-Fe_2_O_3_ was two-fold higher than that of pure g-C_3_N_4_. This improvement in photocatalytic degradation could be attributed to the decreasing electron-hole recombination rate and expanded surface area.

**Figure 7:**
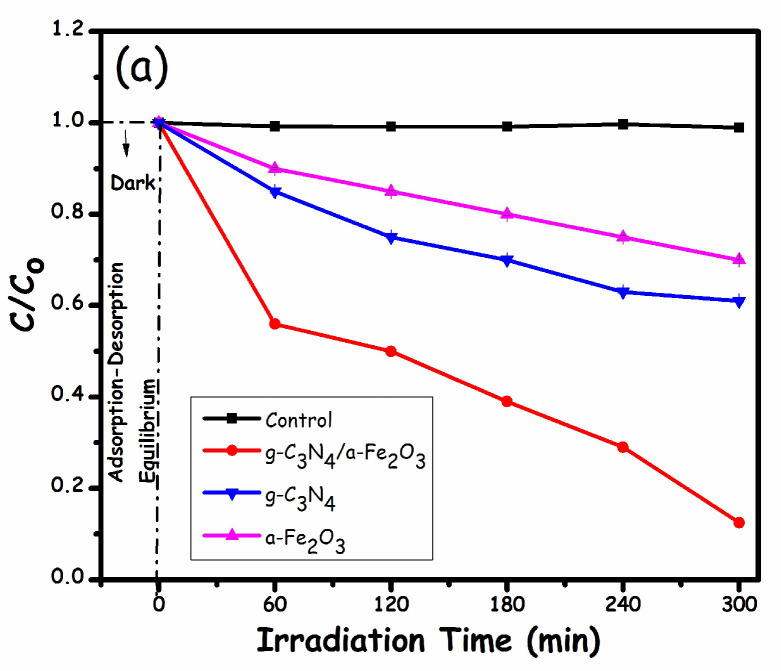

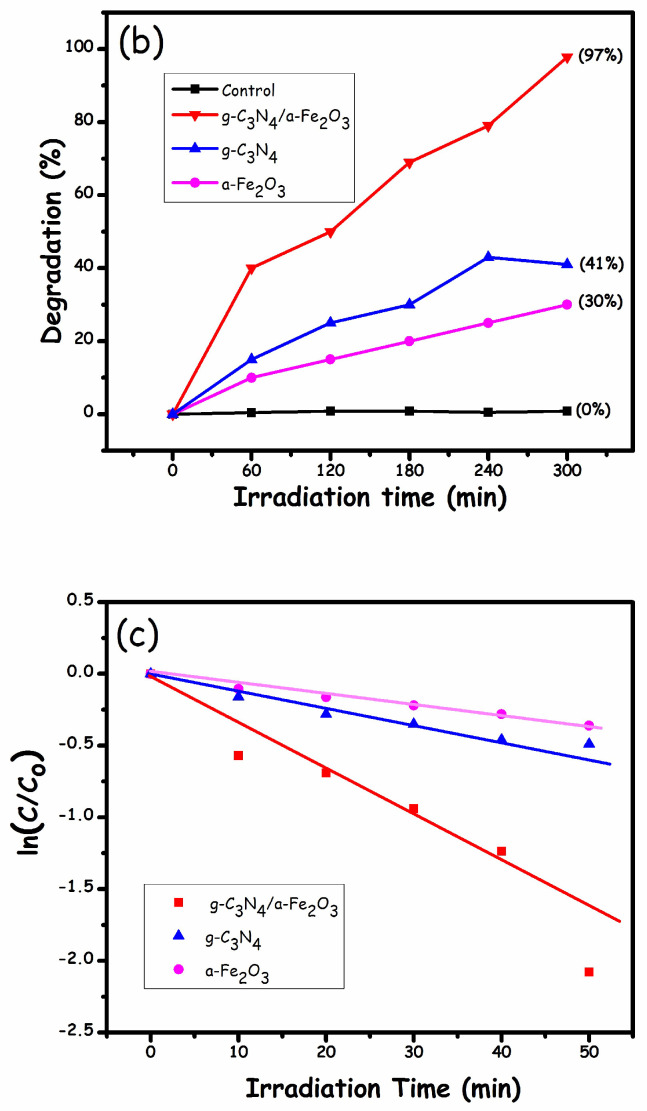
(**a**) Plot of C/C_o_ vs. time. (**b**) The corresponding degradation efficiency of MO removal. (**c**) Plot of ln (C/C_o_) vs. time.

The detailed photocatalytic degradation performance for all the prepared pure and composite photocatalysts was further investigated and is illustrated in Figure 7. A control experiment was also carried out in the absence of a photocatalyst, depicting an almost negligible degradation. Figure 7a represents the change of MO concentration vs. irradiation time under visible light for 5 h. The results revealed that the percentage of MO removal by the g-C_3_N_4_/α-Fe_2_O_3_ nanocomposite was superior, followed by pristine g-C_3_N_4_ and α-Fe_2_O_3_, degrading 97, 41, and 30% of the MO, respectively, as shown in the degradation plot in Figure 7b. The enhanced degradation efficiency of the g-C_3_N_4_/α-Fe_2_O_3_ nanocomposite might be credited to the type-II heterostructure and enhanced surface area. This heterostructure results in enhanced charge carrier separation at the heterojunction interface.

Furthermore, a higher surface area increases the number of available active sites for the absorption and degradation of MO. Moreover, the kinetics of the degradation reaction were also determined, as shown in Figure 7c. The kinetic spectra depict the occurrence of pseudo-first order reactions with all the photocatalysts.

Table 1 summarizes all the prepared photocatalysts’ precise results, including the percentage composition found via EDX, the crystallite size calculated using the XRD values, the bandgap (eV) value ascertained from DRS data, the surface area estimated through BET analysis, and the photocatalytic efficiency.

### 2.3. Photocatalytic MO Degradation Mechanism

**Figure 8:**
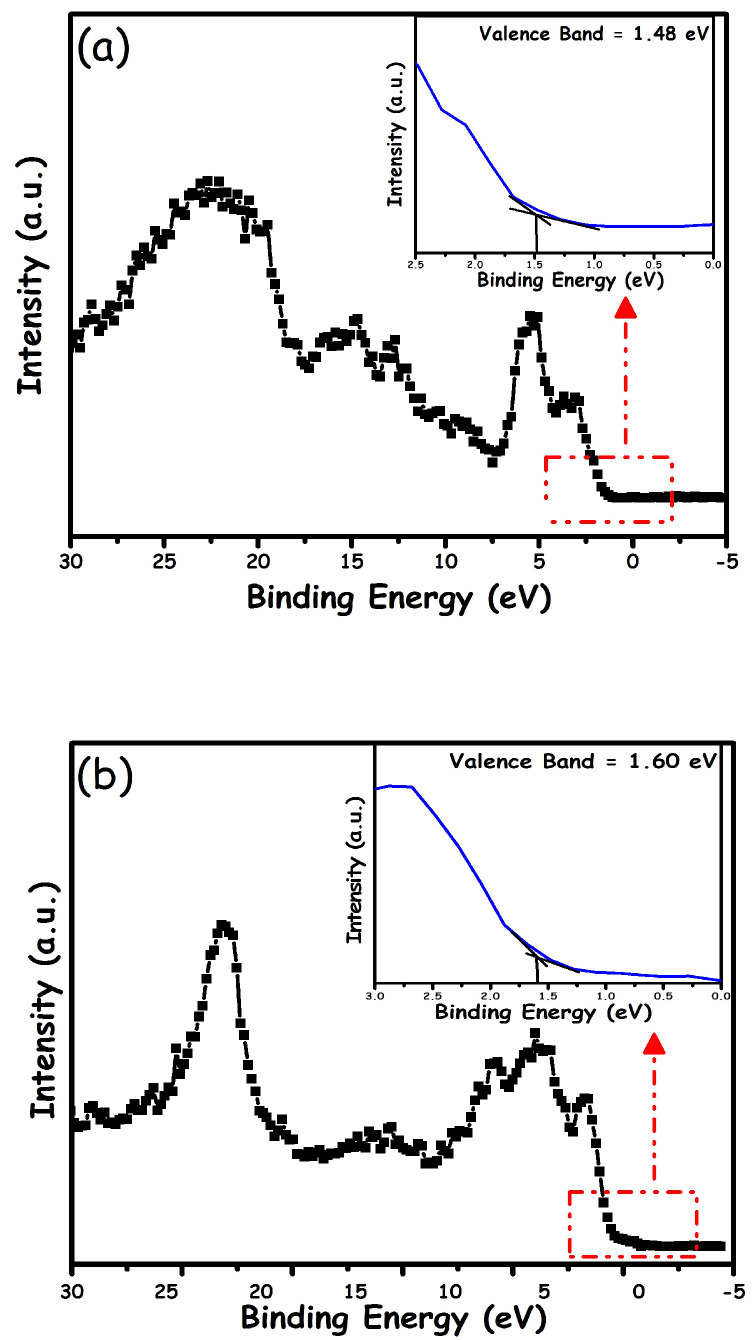

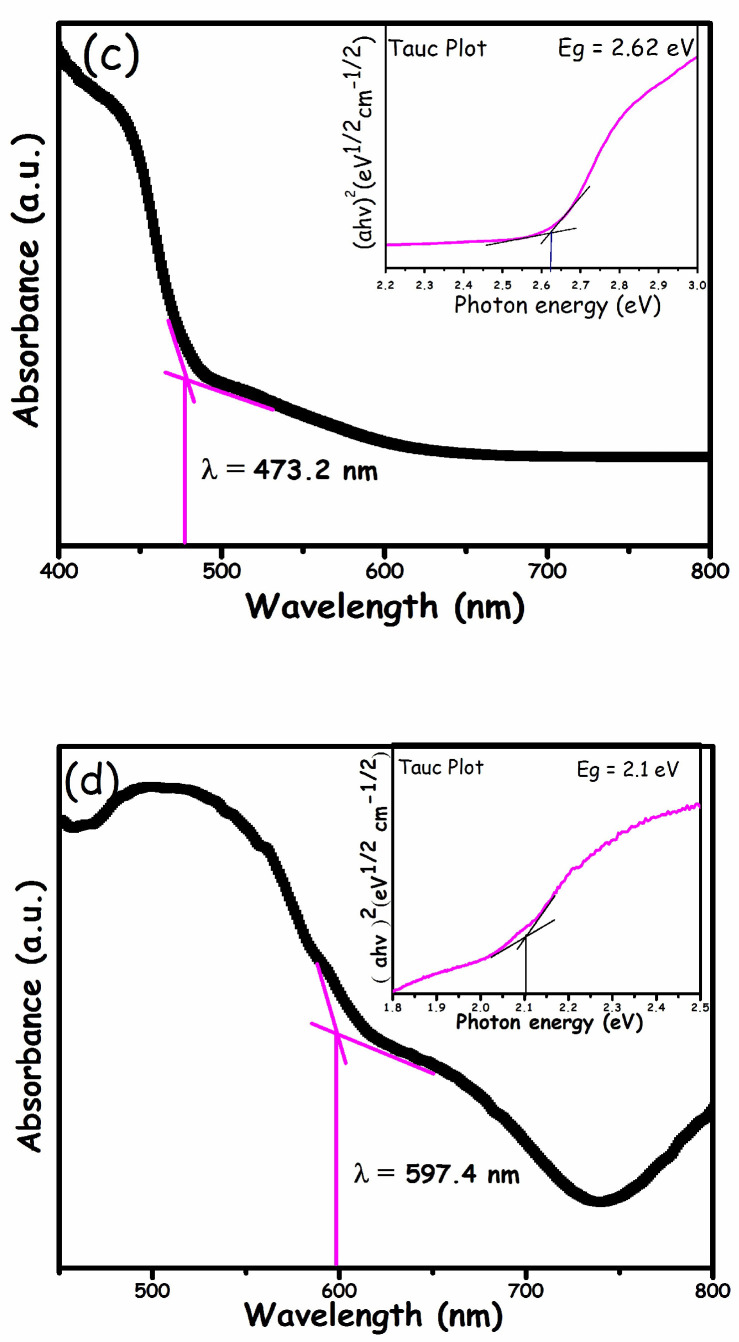
XPS valence band spectra with insets representing magnified spectra of (**a**) g-C_3_N_4_ and (**b**) α-Fe_2_O_3_, and absorbance spectra with insets representing the Tauc plots for (**c**) g-C_3_N_4_ and (**d**) α-Fe_2_O_3_.

The band edge position plays a critical role in determining the reaction mechanism. In this study, XPS was used to calculate the valence band (VB) positions along with UV-visible-DR spectroscopy to ascertain the band gap energies of α-Fe_2_O_3_ and g-C_3_N_4_, and the results are shown in Figure 8. Based on the acquired results, the valence band maximum (VBM) was found to be 1.48 eV and 1.60 eV for g-C_3_N_4_ and α-Fe_2_O_3_, respectively. As the XPS instrument has a work function of 4.62 eV, the final VBM values were estimated to be 1.6 and 1.72 eV against SHE (as 0 V against SHE is equivalent to 4.5 eV against a vacuum) for g-C_3_N_4_ and α-Fe_2_O_3_, respectively. Furthermore, the conduction band minimum (CBM) of the component photocatalysts was calculated using the following equation:

*E*_CB_ = *E*_VB_ − *E*_g_ (1)

**Figure 9:**
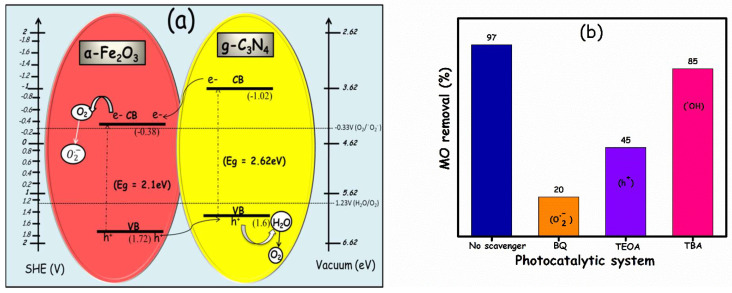
(**a**) Alignment of energy levels in the g-C_3_N_4_/α-Fe_2_O_3_ nanocomposite. (**b**) Role of radical scavengers on MO photodegradation over g-C_3_N_4_/α-Fe_2_O_3_ nanocomposite.

Band gaps (*E*g) were estimated utilizing Tauc plots (Figure 8c,d). Figure 8c displays the DRS spectra of g-C_3_N_4_, showing an optical absorption threshold at 473.2 nm, whereas Figure 8d illustrates the DRS spectra of α-Fe_2_O_3_, showing an absorption edge at 597.4 nm. The band gaps were calculated to be 2.62 eV and 2.1 eV for g-C_3_N_4_ and α-Fe_2_O_3_, respectively, which coincide well with the reported values [40]. Based on these valence band and band gap values, the conduction band values were found to be −1.02 eV vs. SHE for g-C_3_N_4_ and −0.38 eV vs. SHE for α-Fe_2_O_3_. Finally, the energy level diagram was drawn (as presented in Figure 9) utilizing the results from Figure 8.

The alignment of energy levels crucially determines the overall photocatalytic mechanism. When light is turned on, the electrons (e^−^) from the valence band of the components move to their respective conduction bands, leaving behind holes (h^+^), as illustrated in Figure 9. The band edge positions are evidence of the formation of the type-II heterostructure, facilitating the e^−^ and h^+^ transfer from one component to other. In this study, the e^−^ from the conduction band of the g-C_3_N_4_ jumped to the CB of α-Fe_2_O_3_, where the reduction of O_2_ to the superoxide radical anion (O_2_^•−^) takes place. This occurred due to the suitability of the CBM value of α-Fe_2_O_3_ (−0.38 V vs. SHE) with respect to the value of −0.33 V vs. SHE, which is the reduction potential of O_2_/O_2_^•−^. This O_2_ may have been the dissolved O_2_ in the surrounding environment that the experiment was conducted in or the aerobic environment produced as a consequence of H_2_O oxidation. On the other hand, the h^+^ was transferred from α-Fe_2_O_3_ to g-C_3_N_4_. Owing to the more positive band position of g-C_3_N_4_ compared to the H_2_O oxidation potential (1.23 V vs. SHE), the oxidation of water (H_2_O) into oxygen (O_2_) could be taken into account. This antagonistic movement of e^−^ and h^+^ is also responsible for the remarkable enhancement in photocatalytic activity, as it minimizes the chances of their recombination. In previous studies, the photocatalytic mechanism was reported to be driven by different species. For instance, Sangbin lee et al. observed the photodegradation of methylene blue (MB) via O_2_^•−^ over hematite/graphitic carbon nitride composites [44]. Jirong Bai et al. also identified O_2_^•−^ as an active species that further degrades rhodamine B (RhB) over α-Fe_2_O_3_/porous g-C_3_N_4_ [45]. Konstantinos C. Christoforidis et al. illustrated h^+^ as an active species for the degradation of MO over β-Fe_2_O_3_/g-C_3_N_4_ hybrid catalysts, while ∙OH negligibly takes part in the photocatalytic mechanism [46]. Moreover, Xin Liu et al. [47] proposed a similar mechanism, where O_2_^•−^ functions as primary and h+ participates as secondary active species for the degradation of RhB over Fe_2_O_3_/g-C_3_N_4_ photocatalysts. Thus, these reported results are consistent with our study, where we believe O_2_^•−^ plays a primary role in degradation. The primary role of the superoxide radical anion was further attested by the active species trapping experiment results. The trapping experiments were conducted utilizing scavengers for holes (h^+^) and free radicals; the results are presented in Figure 9b. Benzoquinone (BQ), triethanolamine (TEOA), and tert-butyl alcohol (TBA) were utilized as O_2_^•−^, h^+^, and ·OH scavengers, respectively. A control experiment was conducted where no scavenger is utilized, and 97% of MO removal was observed after 5 h of light illumination. The scavenger concentration employed was 0.1 mM. With the addition of BQ, TEOA, and TBA into the solution, the MO removal efficiency was decreased to 20%, 45%, and 85%, respectively. MO degradation was significantly suppressed when BQ was utilized as a scavenger. Thus, the results of the trapping experiments clearly demonstrate that the hydroxyl radical (∙OH) and hole (h^+^) play a minor role in the photocatalytic removal of MO, whereas, O_2_^•−^ is the primary ROS that further degrades the MO over the g-C_3_N_4_/α-Fe_2_O_3_ nanocomposite, which is in good agreement with recent studies [44,45,46,47].

Table 2 demonstrates a comparative study of this work with already reported literature.

### 2.4. Photocatalyst Sustainability

**Figure 10:**
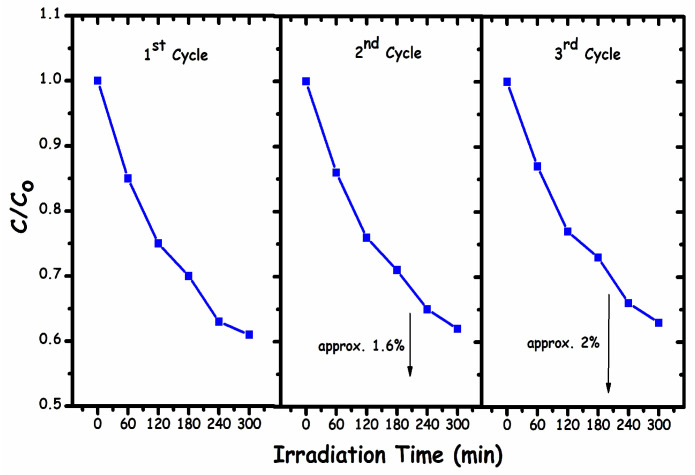
g-C_3_N_4_/α-Fe_2_O_3_ nanocomposite stability.

**Figure 11:**
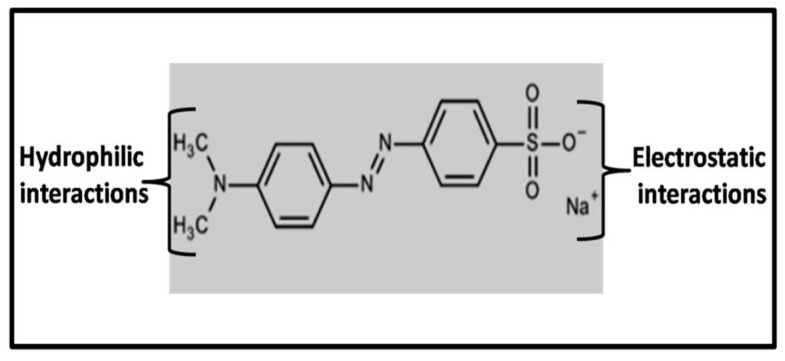
Chemical structure of MO dye.

An essential factor towards the practicability of any photocatalyst is its stability. This work determined the stability of the photocatalyst by recycling the catalyst for three cycles followed by centrifugation and washing with DI-water after each cycle, as illustrated in Figure 10. As shown in the graph, the results revealed a decrement in the degradation efficiency by approximately 1.6% after the first cycle and approximately 2% in the third cycle. This decrease in activity might be ascribed to the loss of photocatalyst during washing and the blocking of active sites for the next run. This is justified by the chemical structure of MO dye (Figure 11), as MO dye adsorbs on the catalyst surface through hydrophilic or electrostatic interactions. When washing the photocatalyst after each photodegradation cycle, there is a chance of the incomplete desorption of the sample from the catalyst’s surface, which leads to the blockage of active sites for the next cycle, thereby decreasing the photocatalytic activity. In addition, the loss of activity might be related to a progressive inactivation of the catalyst (the loss of active phase or catalyst surface modification) [48].

**Table 2:** Comparison with literature.

S. No.	Photocatalysts	Irradiation Source	Time	Conc. of Pollutant and Amount of Catalyst	Pollutant Degraded	Degradation Rate/Efficiency (%)	Ref.
1	Fe_2_O_3_/C_3_N_4_/Au nanocomposite	-	-	MO solution (25 mL, 3 × 10^−3^ M) and 10.0 mg of catalyst	MO	-	Nasri, A. et al. [20]
2	α-Fe_2_O_3_/g-C_3_N_4_ nanocomposite	30 W LED lamp	3 h	MB aqueous solution (2.12 × 10^−5^ M) and 5.5 mg L^−1^ of catalyst	MB	66.79%	Navid Ghane et al. [49]
3	α-Fe_2_O_3_/g-C_3_N_4_ composite	UV lamps (254 nm, 6 W)	90 min	200 mL of 10 mg/L methylene blue solution	MB	2.6 times higher than bare materials	Sangbin Lee [44]
4	α-Fe_2_O_3_/porous g-C_3_N_4_ heterojunction hybrids	500 W Xe arc lamp with 420-nm cut-off filter)	20 min	50 mL of RhB solution and 10 mg/L of catalyst	RhB	91.1%	Jirong Bai et al. [45]
5	ZnO-modified g-C3N4	200 W tungsten lamps	90 min	-	MB	90%	Paul, Devina Rattan et al. [50]
7	Fe_2_O_3_/g-C_3_N_4_ hybrid nanocomposite	300 W Xe arc lamp	4 h	160 mL of aqueous solution containing 10 mg L^−1^ of MO	MO	Approx. 80%	Konstantinos C. Christoforidis [46]
8	g-C_3_N_4_/α-Fe_2_O_3_ nanocomposite	300 W xenon lamp	5 h	0.01 g of catalyst powder in 50 mL dye solution	MO	97%	This work

## 3. Materials and Methods

### 3.1. Chemicals

All the materials and chemicals used for the synthesis were of analytical grade and were used without further purification. Moreover, nanopure water was utilized for the synthesis. Melamine (C_3_H_6_N_6_; >99%), ferric nitrate nonahydrate (Fe(NO_3_)_3_·9H_2_O; >97%), and urea (NH_2_CONH_2_; 99.5%) were purchased from Sinopharm Chemical Reagent Co., Ltd. (Huangpu, Shanghai, China). The model pollutant, i.e., methyl orange (C_14_H_14_N_3_NaO_3_S), was bought from Beijing Chemical Reagent Limited Corporation in Beijing, China.

### 3.2. Preparation of α-Fe_2_O_3_

In a distinctive route, 0.1 M (0.807 g) of ferric nitrate and 0.15 M (0.18 g) of urea were separately dissolved in 20 mL of distilled water and stirred for 15 min. Then, the above two solutions were collectively mixed in a beaker and again stirred for 15 min. The as-prepared mixture was placed in a Teflon-lined sealed autoclave at 100 °C for 8 h. The α-Fe_2_O_3_ was prepared by utilizing ferric nitrate as a source of iron. Afterwards, the sample was washed several times, centrifuged, and dried in a vacuum oven overnight at 60 °C to obtain deep red-colored α-Fe_2_O_3_ nanoparticles. We found that the mixture (ferric nitrate and urea) was highly suitable for the preparation of α-Fe_2_O_3_ nanoparticles. The possible formation mechanism of α-Fe_2_O_3_ nanoparticles involves a series of chemical reactions (Equations (2)–(4)).

The importance of utilizing urea lies in the fact that for the synthesis of hematite, the basic source and its dosage affect the morphology, e.g., a small amount of urea produces a less hollow and more interconnected morphology while a large amount of urea produces solid hollow microspheres [51]. Upon heating to 70 °C, the dissolved urea decomposes to carbon dioxide and ammonia (Equation (2)). Carbon dioxide (CO_2_) bubbles produced during the hydrolyzation play an important role. CO_2_ acts as a soft template for the formation of the hollow structure. In our study, since the dosage of urea was only 0.18 g, the amount of CO_2_ produced was too minute to form the microbubbles under that situation. Therefore, a more interconnected morphology of the nanoparticles of hematite was obtained. Furthermore, the hydrolysis of ammonia yields the ammonium ion and hydroxyl ion (Equation (3)). The hydroxyl ion reacts with a ferric ion and generates Fe(OH)_3_, which is the primary growth nucleus with an amorphous structure. The further combination and growth of neighboring primary nuclei leads to the formation of agglomerated hematite NPs [52] (Equation (4)).

(2) $${\mathrm{CH}}_{4}N_{2}O+{\;H}_{2}O\to {\mathrm{CO}}_{2}+2{\mathrm{NH}}_{3}$$

(3) $${\mathrm{NH}}_{3}+H_{2}O\;\to {\mathrm{NH}}_{4}^{+}+{\;\mathrm{OH}}^{-}$$

(4) $${\mathrm{Fe}}^{3+}+{\;\mathrm{OH}}^{-}\to \mathrm{Fe}{\left(\mathrm{OH}\right)}_{3}\to \;\alpha -{\mathrm{Fe}}_{2}O_{3}$$

### 3.3. Preparation of g-C_3_N_4_

The g-C_3_N_4_ was synthesized through a simple calcination approach by placing 5 g melamine in a ceramic crucible followed by heating in a muffle furnace at 550 °C at a 3 °C/min ramp rate for 2 h. The resulting yellow-colored g-C_3_N_4_ precipitates were stored for further experimental use.

### 3.4. Preparation of g-C_3_N_4_/α-Fe_2_O_3_

The synthesis procedure for the fabrication of the g-C_3_N_4_/α-Fe_2_O_3_ composite is identical to α-Fe_2_O_3_ synthesis. Before heating, 0.2 g of as-synthesized g-C_3_N_4_ was added to the reaction mixture of ferric nitrate and urea followed by ultrasonication for 60 min. Subsequently, the mixture was placed in oven at 100 °C for 8 h. Afterward, the sample was washed with ultrapure water and then with ethanol, followed by centrifugation and drying in a vacuum oven overnight at 60 °C. Finally, the light red colored precipitates were obtained and stored for further characterization.

### 3.5. Characterization Techniques

The phase purity and structural analysis of as-synthesized photocatalysts were studied by X-ray diffraction spectroscopy (XRD, Cu Kα radiation, Bruker D8) with 2θ range 20°–80° at a rate of 0.1 °C/min. In addition, the crystallite size of all the catalysts was calculated using Scherrer’s formula:

(5) D=Kλ/βcosθ 

where K is the shape constant with a value of 0.89, D represents the crystallite size, λ is wavelength (Cu k-alpha generally has a wavelength of 0.15405 nm), β is the full width at half maxima (FWHM) of the observed peak, and θ represents the angle. The morphological analysis was carried out by scanning electron microscopy (SEM; Hitachi S4800), equipped with energy dispersive X-ray spectroscopy analysis, which further justified the samples’ composition and purity. The chemical states and valence band positions were determined by X-ray photoelectron spectroscopy (XPS; ESCALAB 250Xi, Al Kα). A UV-vis spectrophotometer (UV-750, Indium as reference) was employed for DRS reflectance spectra. The DRS spectra were transformed to absorption spectra by utilizing the Kubelka–Munk equation:

(6) $${\;\alpha h\nu }^{1/2}=K\left(h\nu -\mathrm{Eg}\right)$$

where α represents the absorption coefficient, K is the proportional constant, h represents Plank’s constant, ν is the vibration frequency, and Eg represents bandgap (eV). Additionally, the BET specific surface area was measured using the nitrogen adsorption–desorption method at 77K (BET, BELSORP-mini II).

### 3.6. MO Degradation Activity

Methyl orange (MO) was used as a model dye, and its photodegradation response was investigated by utilizing a UV-vis spectrophotometer. The degradation activity was carried out by dispersing 0.01 g of catalyst powder in dye solution with a concentration of 100 ppm. Prior to light exposure, the suspension was placed aside for 20 min in order to develop the adsorption–desorption equilibrium. MOmax was observed at 464 nm. Eventually, the light source was turned on. The light source was a 300 W xenon lamp (number of the lamp was 2) with a cut-off filter (>420 nm) with an output power density of 100 mW/cm^2^, which was placed at a distance of 15 cm from the vessel containing the dye solution. Three milliliters of the irradiated suspension were collected at various time intervals, followed by centrifugation to analyze the dye concentration. Moreover, a control experiment was also carried out in light but without any catalyst and is labeled as control in the photocatalytic degradation plots.

## 4. Conclusions

The domain of developing nanocomposites to stop recombination for a higher photocatalytic response has already been well established. The novelty of this study lies in its investigation of the mechanistic route and type of reactive oxygen species involved in the photodegradation of MO, utilizing a g-C_3_N_4_/α-Fe_2_O_3_ nanocomposite. Based on the results of the characterization assays, the energy level diagram and suitable band edge positions vs. SHE justify this study. The g-C_3_N_4_/α-Fe_2_O_3_ nanocomposite showed a superior photocatalytic response towards the photodegradation of MO than its individual counterparts. The redox potential values with respect to the valence and conduction band values suggest a potentially heterojunction-based photocatalyst mechanism. Additionally, the kinetics of the degradation reactions were also monitored. Moreover, the photocatalyst sustainability experiment depicted the practical application of this nanocomposite. In the future, the potentially potent g-C_3_N_4_/α-Fe_2_O_3_ nanocomposite can be utilized for other photocatalytic applications, including CO_2_ reduction or photoelectrochemical studies.

## Figures and Tables

**Table 1:** Summary of all characterization techniques.

S#	Sample Code		EDX—Percentage Composition			XRD—Avg. Crystallite Size (nm)	DRS—Band Gap (eV)	BET—Surface Area (m^2^/g)	Photocatalytic Efficiency (%)
		Atomic % of C	Atomic % of N	Atomic % of O	Atomic % of Fe				
1	g-C_3_N_4_	36.86	63.14	----	----	29.4	2.62	39.89	41
2	α-Fe_2_O_3_	----	----	60.36	39.64	32.5	2.1	34.25	30
3	g-C_3_N_4_/α-Fe_2_O_3_	9.81	17.05	23.20	49.94	60.5	----	80.38	97

## Data Availability

Not applicable.
